# Characterization, costs, cues and future perspectives of phenotypic plasticity

**DOI:** 10.1093/aob/mcac087

**Published:** 2022-06-30

**Authors:** Hannah M Schneider

**Affiliations:** Centre for Crop Systems Analysis, Wageningen University & Research, Wageningen, the Netherlands

**Keywords:** Phenotypic plasticity, environmental cues, genotype x environment interaction, phenotypic variation

## Abstract

**Background:**

Plastic responses of plants to the environment are ubiquitous. Phenotypic plasticity occurs in many forms and at many biological scales, and its adaptive value depends on the specific environment and interactions with other plant traits and organisms. Even though plasticity is the norm rather than the exception, its complex nature has been a challenge in characterizing the expression of plasticity, its adaptive value for fitness and the environmental cues that regulate its expression.

**Scope:**

This review discusses the characterization and costs of plasticity and approaches, considerations, and promising research directions in studying plasticity. Phenotypic plasticity is genetically controlled and heritable; however, little is known about how organisms perceive, interpret and respond to environmental cues, and the genes and pathways associated with plasticity. Not every genotype is plastic for every trait, and plasticity is not infinite, suggesting trade-offs, costs and limits to expression of plasticity. The timing, specificity and duration of plasticity are critical to their adaptive value for plant fitness.

**Conclusions:**

There are many research opportunities to advance our understanding of plant phenotypic plasticity. New methodology and technological breakthroughs enable the study of phenotypic responses across biological scales and in multiple environments. Understanding the mechanisms of plasticity and how the expression of specific phenotypes influences fitness in many environmental ranges would benefit many areas of plant science ranging from basic research to applied breeding for crop improvement.

## INTRODUCTION

The sessile nature of plants requires them to respond effectively to their constantly changing environment. Plants have developed highly sophisticated and efficient strategies to avoid, tolerate and adapt to challenges of unfavourable growth conditions, diseases, herbivores and other abiotic and biotic stresses. These plastic responses are found across all taxa and are a defining feature of life (Nijhout, 2003). All organisms can modify their phenotype or internal physiology to maintain a stable equilibrium (Sultan, 2015). These responses can vary in duration and magnitude and involve changes in morphology, resource allocation, anatomy, physiology or development. Plastic responses range from conspicuous changes in leaf area to subtle changes in gene or transporter expression and often are not (directly) visible to the naked eye.

All organisms are plastic because one or more of their traits respond to the environment. However, given that nature is typically a heterogeneous environment, why is not every organism plastic for many traits, and why are not most or all species adapted to a broader range of environments? Many species display a range of plastic responses across a range of phenotypic traits at different scales; however, adaptive plastic responses are limited and dependent on the trait and environment. Challenges in measuring, characterizing and interpreting plastic responses and their costs and limits have hindered our understanding of adaptive and maladaptive plastic responses and to what extent they have evolved or become fixed.

Many existing reviews focus on phenotypic plasticity and describe terminology, molecular mechanisms, and the role and influence of plasticity in evolution and selection (e.g. Sultan, 2000; Givnish, 2002; Grime and Mackey, 2002; Novoplansky, 2002; Sachs, 2002; Schlichting and Smith, 2002; West-Eberhard, 2003; Nicotra *et al.*, 2010; Kelly *et al.*, 2012; Pfennig, 2021). This review highlights emerging ideas and approaches in plasticity research and their application in plant science. The field of plasticity is broad, and transdisciplinary ideas and approaches are needed to understand plant growth and development. First, the characteristics and descriptors of phenotypic plasticity in plants are addressed. I argue the need to use more precise descriptors to describe plastic responses when working across biological scales on a wide range of species. Next, I draw attention to the importance of reliable environmental cues for the adaptive value of phenotypic plasticity. In addition, I address the costs of plasticity and how these costs fit into the cost–benefit paradigm of expressing plasticity. Finally, I focus on promising approaches and new research directions in studying phenotypic plasticity. Phenotypic plasticity is a broad field of research; however, linking approaches and ideas across species, systems and disciplines can benefit many domains of plant science ranging from basic research to crop breeding.

## CHARACTERISTICS OF PLASTICITY

There is a wide range of terminology used to describe phenotypic plasticity. The literature contains several definitions of plasticity, which centre around the key theme of phenotypic responses of an organism associated with different environments (Stearns, 1989; Piersma and Drent, 2003; West-Eberhard, 2003). However, the concept of plasticity is deceptively simple. The word ‘plasticity’ can be confusing as it does not refer to any obvious characteristic of living organisms. A species or genotype cannot be characterized as entirely plastic. While the type, duration and degree of plastic responses are a property of genotypes, in most plants, plasticity is expressed at the level of modular subunits (i.e. semi-autonomous structural and functional components of plants including leaves, roots and individual meristems) (De Kroon *et al.*, 2005). For a specific genotype, an individual trait may have a plastic response to a specific environment, but a canalized response (i.e. a phenotype expressed consistently across a specified environmental range) to other environmental factors or factor levels, and it may express plasticity in some traits but not others. Phenotypic plasticity is a response to fine-grained heterogeneity at a structural or physiological level rather than the functional individual (De Kroon *et al.*, 2005). These localized modular responses have been described in many studies, including local proliferation of lateral root growth in response to a nutrient patch (Drew *et al.*, 1975), the development of more and larger buds on tree branches grown in sunny patches when compared to shaded patches, or in shade conditions the development of leaves with morphological and physiological properties that enhance light capture and photosynthetic efficiency (Sprugel *et al.*, 1991). However, modules can be interconnected, and communication between modules also may influence plastic expression. For example, communication between modules (e.g. via hormones) may enhance or reduce local plastic effects and increase or decrease the differences between integrated modules exposed to different environmental conditions (De Kroon *et al.*, 2005). Many examples of integration of modules in response to environmental cues are provided in the literature and are apparent in roots, branches, leaves and other organs (e.g. Sprugel *et al.*, 1991; Robinson, 1994; Grime and Mackey, 2002). Furthermore, canalization is also important to consider as the canalization of a specific trait in the face of environmental cues often requires plastic expression in another trait. The measurement of phenotypic plasticity on a whole plant scale is the sum of all the environmentally induced modular responses and interaction effects between modules (De Kroon *et al.*, 2005).

The quantification and interpretation of plastic responses can be considered on a broad range of biological scales ranging from genes, traits and even between species (e.g. Carroll and Corneli, 1991; Thaler and Pages, 1996; Karban and Nagasaka, 2004; Nussey *et al.*, 2007; Lachowiec *et al.*, 2016). Therefore, several characteristics can be used to describe specific phenotypic responses. The timing, duration, adaptive value, inheritance and other factors (described below) can describe and define specific responses (Table 1). Many of these factors are not exclusive, and often several can be used to define responses to a specific trait, genotype and environment combination. It is important to note that the biological scale on which plastic responses should be measured is an ongoing source of ambiguity and discussion in the literature. Here, plasticity refers to responses at all scales, from genes and proteins to morphological changes in organs.

**Table 1.** Characteristics of phenotypic plasticity. Table modified from Pfennig (2021).

Canalized or plastic trait (general)	Canalized: consistent phenotype across an environmental range	Plastic: phenotypic response to an environmental cue
Short or long duration	Short duration, labile: the trait can change in response to the environment	Long duration, fixed: the phenotype cannot change once established
Active or passive	Active: a ‘switch’ occurs in the metabolic or developmental system; anticipatory; autoregulatory morphogenesis	Passive: trait responds by a general shift; dependent morphogenesis; responses measured on allocation traits or plant age; typically due to resource limitations
Instantaneous or delayed	Instantaneous: phenotypic response to the environmental signal occurs instantly	Delayed: there is a time lag between environmental signal and phenotypic response
Genetic vs. non-genetic inheritance	Genetic: inherited traits are passed from parent to offspring according to Mendelian genetics	Non-genetic: involves the influence of ancestors on descendants that are not mediated by genetic allele transmission
Continuous or discrete	Continuous: trait can be described as a reaction norm and displays a range of phenotypes rather than discrete	Categorical: trait exists in two or more discrete forms
Reversible or irreversible	Reversible: phenotypic change reversed upon exposure to non-inducing environment	Irreversible: phenotypic change remains fixed upon exposure to non-inducing environment following an inducing environment
Adaptive or maladaptive	Adaptive: enhances fitness of the individual	Maladaptive: does not enhance fitness of the individual
Cryptic or limited	Cryptic: the plastic response of the trait is not expressed in the range of conditions in the ancestral environment	Limited: small range of plasticity expressed in the ancestral environment

### Adaptive and maladaptive plasticity

Many plastic responses increase the organism’s fitness, a phenomenon known as adaptive plasticity. There are numerous examples of adaptive plastic responses, including reduced root secondary growth in common bean as a response to phosphorus stress (Strock *et al.*, 2018) or a faster rate of stomatal closure in response to drought (Wu *et al.*, 2021). However, the adaptive value of plastic responses is dependent on the environment, trait and its interaction with other traits. For example, reduced secondary growth in common bean may also promote more arbuscular mycorrhizal associations compared to roots with greater secondary growth, thereby additively increasing its adaptive value under phosphorus stress. However, reduced secondary growth may not be adaptive in all environments and for instance may increase the plants’ susceptibility to infestation by pathogens and diseases (Strock *et al.*, 2018; Strock and Lynch, 2020). Therefore, the interpretation of adaptive plastic responses must be carefully considered. A recent meta-analysis estimated that approximately one-third of studies potentially misinterpret plastic traits associated with adaptive or functional responses (Bonser, 2021). In addition, several other meta-analyses indicated that plasticity does not always affect fitness, nor is it always adaptive (Van Buskirk and Steiner, 2009; Davidson *et al.*, 2011; Palacio-López *et al.*, 2015; Acasuso-rivero *et al.*, 2019). Plasticity in functional or fitness traits does not indicate an adaptive response. Distinguishing the interpretation of plasticity in functional or physiological traits versus performance traits is essential (Bonser, 2021).

Many authors imply that plastic responses are inherently adaptive (emphasizing how past selection events shaped these responses); however, not all plastic responses are adaptive in all environments, particularly in fluctuating environments with multiple, simultaneous dynamic stresses. Fluctuating and unpredictable environments may deem once adaptive plasticity maladaptive as new, unpredictable environments cause mismatches between the phenotype and environment. For example, lateral root proliferation in response to nutrient patches may be an adaptive strategy for enhanced nitrogen acquisition (Mi *et al.*, 2010); however, if the nitrate moves faster through the soil profile than roots proliferate, this response may be deemed maladaptive (Schneider and Lynch, 2020). The adaptive value of plastic responses for plant fitness is trait-specific and depends on several environmental factors.

### Active and passive plasticity

Active plasticity is generally anticipatory, highly integrated, and results in responses to an environmental signal that may involve modification in developmental pathways or regulatory genes (Forsman, 2015). In contrast, passive plasticity (i.e. apparent plasticity) may result from resource limitations, allometry or ontogeny and is generally not anticipatory but a mere consequence of the environment (Weiner, 2004; Forsman, 2015) (Table 1). For example, in environments with low soil nitrogen availability, generally, above- and below-ground biomass and the quantity and quality of reproductive organs will be reduced due to nitrogen limitation (Naegle *et al.*, 2005). However, this reduced growth in response to the environment is a type of passive plasticity as it is a consequence of inevitable resource limits and physical conditions.

In addition, many species alter their biomass allocation patterns during ontogeny, and often traits have strong allometric associations. Therefore, environmental factors influencing development or growth rates may also influence biomass partitioning and allometry. Depending on the developmental stage, environmental cues may trigger qualitatively and quantitatively different responses, termed ‘ontogenetic contingency’ (Diggle, 1994; Watson *et al.*, 1995). For example, not all floral primordia are plastic within inflorescences of *Solanum hirtum*, an andromonoecious woody perennial. Only flowers developing at the distal positions in each inflorescence have the ability to be plastic by altering gynoecial development; primordia initiated at basal positions are invariably hermaphrodite. Therefore, plasticity at the flower level varies ontogenetically with the development of new inflorescences (Diggle, 1994).

Another example is demonstrated by changes in root to shoot ratios. Changes in root to shoot ratios are associated with nutrient deficiencies (Poorter and Nagel, 2000; Ho *et al.*, 2005), soil compaction (Atwell, 1993), drought (Huang and Fry, 1998) and other abiotic stresses. Changes in root to shoot ratios are often explained by the functional equilibrium theory, or the allocation of biomass to roots or shoots depending on the availability of above- or below-ground resources to prioritize and optimize the acquisition of resources in a manner that maximizes plant growth (Poorter and Nagel, 2000). For example, increases in root to shoot ratios in response to low nitrogen availability typically occur as long as the availability of assimilates is not limiting (Ericsson, 1995). In conditions of nutrient limitation, large amounts of carbon may be allocated to root growth to obtain the most limiting resource, which may be considered an adaptive response (Poorter and Nagel, 2000). However, younger or smaller plants generally have a greater root to shoot ratio, and ontogeny may explain this plastic response if we consider these plants to have reduced growth rates or lag developmentally. Independent of environmental conditions, there may be a pre-defined root to shoot ratio for each plant size. Therefore, the root to shoot ratios may reflect the smaller plant size, a form of passive plasticity, and not an adaptive response to stress (Correa *et al.*, 2019). Plastic responses and phenotypic expression pathways are governed by developmental and phylogenetic constraints and natural selection, and therefore the expression of a phenotype in a specific environment may reflect both active plastic responses and developmentally inevitable aspects of plant growth (Sultan and Stearns, 2005; Martin and Isaac, 2021).

### Plasticity of short and long duration

Plastic responses can also vary in duration, ranging from more permanent responses (i.e. long-duration plasticity) to physiological plasticity (i.e. short-duration plasticity). Short-duration plasticity may enable plants to respond to temporally variable aspects of the environment, including light intensity or water and nitrate availability. For example, aquaporin expression can fluctuate rapidly due to water availability (Zargar *et al.*, 2017). In contrast, plasticity of a longer duration may involve changes due to morphological or developmental plasticity. For example, the number and size of cells in the root cortex are established near the growing root apex, and the potential for change in many mature tissues is limited. However, the threshold between short- and long-duration plasticity in many cases is unclear and varies by trait, genotype and species.

### Reversible or irreversible plasticity

Reversible (i.e. phenotypic flexibility) and irreversible plastic responses also describe plasticity of variable duration that can change along the growing apices upon stress release. A short-term stress exposure may induce a plastic response for the lifetime of the plant (whether or not the stress continues) (i.e. irreversible), or the plastic response may no longer be actively developed when the stress is released (i.e. reversible). For example, soil hypoxia or increased penetration resistance reduced root elongation in pea and wheat. However, upon stress release, pea roots were able to recover, and the elongation rate was accelerated upon stress release, demonstrating reversible plasticity. Wheat exhibited an irreversible plastic response, and the elongation rate remained retarded after stress release (Sjulgård *et al.*, 2021). In fluctuating environments, it is interesting to measure the direct response to stress and how plastic responses change (or do not change) after stress release. Genotypic or species variation in reversible and irreversible plasticity of traits may be an important source of variation for understanding the adaptive value of plasticity and its costs. However, it is important to consider the modularity of plants and plastic expression when interpreting the reversibility of plastic responses and other characteristics. For example, when wheat roots were no longer exposed to hypoxia or increased penetration resistance stress, their root elongation rates remained retarded; however, at the plant level, subsequent modules may express different plastic responses that may be reversible. Presumably, the modular subunits of plants can express different types of plastic responses to the same environmental signals (Table 1).

The term ‘developmental plasticity’ is often used synonymously with irreversible plasticity. However, there are several examples of organisms that undergo what is presumed to be permanent, irreversible developmental plasticity, but nevertheless, specific environmental conditions later in their life cycle can revert or modify these plastic responses. Examples documented in plants are rare (although presumably, they occur), but they have been well documented in animals. Developmental switching of mouth formation in nematodes, spinal cord organization in zebrafish and cold tolerance in fruit fly are all responses of long or presumed permanent duration that can be reverted to their original state much later in development (Slotsbo *et al.*, 2016; Werner *et al.*, 2017). These ‘corrected’ or ‘reverted’ phenotypes differ from truly reversible ones as they may not involve a true reversal (backtracking) along a developmental pathway (Burggren, 2020). Critical developmental windows in which traits may be sensitive to environmental cues represent an important yet overlooked concept in plasticity research.

In the literature, there is discussion on how acclimation fits into the current framework of phenotypic plasticity, its assumed inherent adaptive value and its synonymous use with reversible plasticity (see references in Woods and Harrison, 2002; Deere and Chown, 2006; Fenollosa and Munné-Bosch, 2019; Burggren, 2020). Acclimation is often broadly defined as the gradual change of an organism in response to its environment that is reversible and repeatable in the lifetime of individuals (Utz *et al.*, 2014). Acclimation may refer to a type of plasticity with a specific duration, timing, scale and quantitative nature. However, it is not only defined by its reversibility. Some authors have even differentiated between irreversible ‘developmental acclimation’ and reversible ‘short-term acclimation’ in terms of cold hardening responses in insects (Noh *et al.*, 2017). Here and throughout the review, examples are provided of acclimation under the broader context of plasticity and not only defined by its potentially reversible nature.

### Instantaneous or delayed plasticity

The response time between an environmental cue and phenotypic expression of a plastic response is also important. Generally, phenotypes with a shorter lag time (i.e. shorter time between the environmental cue and induction of plasticity, relative to the environmental variability) are more adaptive (in terms of fitness) than responses with a longer lag time (Padilla and Adolph, 1996). Delayed adaptive plastic responses to a specific environment may no longer be adaptive if the environment has changed by the time the organism has adjusted its phenotype (Levins, 1968). Lag times between the environmental cue and expression of phenotypic plasticity have been observed in several species, including the induction of chemical defences in marine algae or timely stomatal closure in response to drought (Van Alstyne, 1988; Harvell and Padilla, 1990; Martin-StPaul *et al.*, 2017). Brown algae that had been grazed increased their concentrations of polyphenolic compounds by ~20 % compared to uninjured plants. However, the response time was variable among individuals and could take up to 2 weeks after injury (Van Alstyne, 1988). The length of the lag time may have large effects on the fitness of the plastic response, as longer lag times generally increase the probability of mismatches between the environment and phenotype.

### Continuous and discrete plasticity

Phenotypic plasticity can be expressed both continuously and discretely. Plasticity expression on a continuous scale is more common than on a discrete scale (i.e. polyphenism). Plasticity expressed continuously may enable individuals to tune their response to the strength and duration of the environmental stimulus. For example, stomatal conductance is decreased relative to the duration of waterlogging in wheat. Wheat plants exposed to waterlogging for a longer duration had greater decreases in stomatal conductance compared to plants exposed to waterlogging for a short duration (Herzog *et al.*, 2016). Examples of discrete plastic responses in plants include seasonal forms, alternative reproductive forms and heterophylly (i.e. different leaf forms on the same plant) (Wells and Pigliucci, 2000). However, in the case of several polyphenisms, an individual may express environmental robustness in a range of environments but, in response to repeated environmental cues or different environmental ranges, may express plasticity for a specific polyphenism (Bateson and Gluckman, 2011). For example, a common polyphenism in flowering plants is cleistogamy, the phenomenon where the same plant may produce open, cross-pollinated flowers as well as highly reduced, closed, self-pollinated (cleistogamous) flowers. Cleistogamous flowers are not produced in a range of environmental conditions, including large ranges of light intensity, but may be induced by specific low levels of light (Joly and Schoen, 2021).

### Genetic and non-genetic inheritance of plasticity

A more complex state of plasticity is non-genetic inheritance. In the broadest sense, non-genetic inheritance involves the influence of ancestors on descendants that are not mediated by genetic allele transmission. A subset of non-genetic inheritance includes parental effects or provisioning. For example, the amount and composition of tissues provisioned by the parent to the seed often reflect resource availability to the parent and subsequent effects on the size or growth rate of the offspring (Fenner and Thompson, 2005). Non-genetic inheritance also encompasses transgenerational plasticity, which involves non-genetic transmission of phenotypes induced by a specific environment (Bell and Hellmann, 2019).

The expression of many phenotypic traits can be influenced by transgenerational plasticity, including morphology and fitness-related traits (Bell and Hellmann, 2019). For example, in several species, the expression of defensive traits can be induced by predator cues in individuals that experience the cues directly and in their offspring (Colicchio, 2017). These transgenerational plastic responses will often induce effects in the offspring (or in some cases grand offspring) that are similar to the direct effects induced in the parent (Kathiria *et al.*, 2010). However, there are cases in which the direct effect of the environment on an individual may be different from its effect on its offspring (Galloway and Etterson, 2007).

To understand plasticity, we need to better define the response, including if the response is active or passive, continuous or discrete, and adaptive or maladaptive (Table 1). Similar terminology and concepts to those described above are commonly used in different phenotypic dimensions, including morphology, physiology, life history, and when the phenotype changes in response to variation or a cue in the external or internal environment. This may be a strength, as the field of plasticity may benefit from common, unifying theoretical frameworks. Given the vast terminology in the field of plasticity (see examples in Nicotra *et al.*, 2010; Forsman, 2015), researchers should take care of how they use terminology, characterize plasticity and interpret results, as broad concepts and words can be misleading.

## ENVIRONMENTAL CUES

Plastic responses involve two stages: assessment and interpretation of environmental cue(s) and the subsequent response (West-Eberhard, 2003; Sultan, 2015). Environmental information is assessed and the organism must ‘decide’ how to use that information to respond, or not respond, to express the phenotypes that will most probably yield the highest fitness. Organisms that can gather, assess and respond to available information to evaluate environmental signals accurately should have greater fitness. However, how plants assess and process environmental information is often overlooked in research, probably due to the dynamic complexity of these mechanisms.

**Fig. 1.**
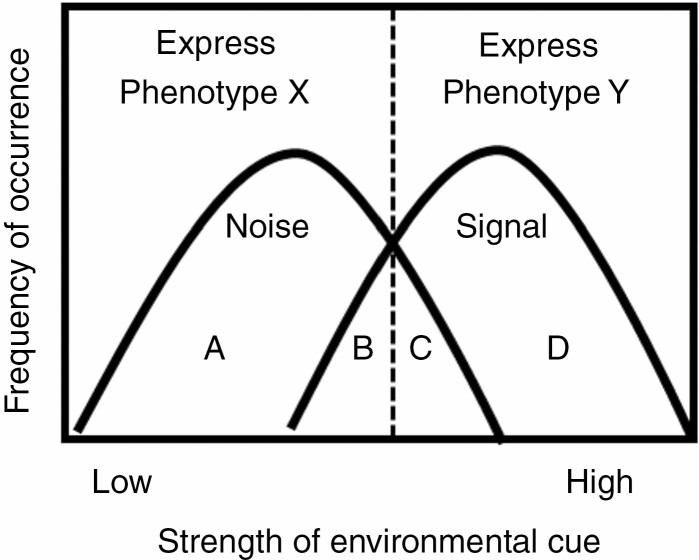
Signal versus noise detection. Genotypes must discriminate between relevant (cue) and irrelevant (noise) environmental information. In general, there is a threshold value of the strength of the cue (dashed lines) in which the organism will express plasticity (in this case, phenotype X or Y) depending on if it is perceived as a signal or noise. The optimal location of the threshold shifts depending on the costs and benefits of correctly responding to a signal (D), incorrectly responding to a signal (B), incorrectly responding to noise as a signal (C) or correctly not responding to noise (A). Modified from Pfennig (2021).

Several signal detection and threshold theories suggest how organisms detect signals (i.e. relevant information for plant fitness) from noise (i.e. irrelevant information for plant fitness). For example, the ‘signal detection theory’ highlights how the response threshold (i.e. the threshold at which the organism produces one phenotype as opposed to another) should be maintained so that the organism is sensitive enough to correctly detect and respond to relevant signals, but not too sensitive as to respond too frequently to noise (Wiley, 2015). In the ‘acceptance threshold theory’, the optimal response may be when the signal and noise overlap, but when the benefits of responding to the signal outweigh the costs of failing to respond to the signal (or incorrectly responding to the noise) (Fig. 1). The ‘acceptance threshold’ may shift depending on the benefits and costs of these responses (Reeve, 1989; Moczek and Nijhout, 2003). For example, once trigger hairs are stimulated on a Venus flytrap, it must ‘decide’ whether the encaged object is potential food and whether or not it should activate glands to produce prey-degrading hydrolases. The trap will close after two trigger hairs are stimulated. However, more than three trigger hairs are required to be stimulated to produce prey-degrading enzymes. The amount of prey-degrading enzymes produced is proportional to the number of mechanical stimulations (Böhm *et al.*, 2016). The greater number of mechanical stimulations increases the likelihood of a large insect encaged whose benefit to the Venus flytrap should outweigh the costs associated with producing the decomposing enzyme cocktail. However, depending on the variability of the environment and the cost–benefit ratio of the plastic response, fitness may be maximized by expressing an optimal, limited degree of plasticity, which may minimize large phenotypic mismatches but avoids the potentially high cost of plastic responses (Haaland *et al.*, 2021).

### Availability and reliability of environmental cues

Climate change is expected to influence the availability and reliability of environmental cues. First, climate change may disrupt the detection of cues by degrading the signal or disturbing the organisms’ sensory abilities. This phenomenon is more extensively studied in animals when compared to plants. For example, eutrophication can impair visual signals for male sticklebacks, and their increased investment in courtship behaviour can become maladaptive (Lonnstedt *et al.*, 2013). In addition, dead coral reefs no longer provide predator cues for juvenile damefish (Candolin, 2009). In plants, UV radiation can damage photosynthetic machinery (Bornman *et al.*, 2019), which may alter stomatal conductance and other traits. In cases when the environmental cue is still available, the reliability of the cue can be influenced if the novel environment is in some aspects similar to a known environment, but not correlated with the selection environment (i.e. evolutionary or ecological traps). In addition, the correlation between the cue and the selective environment may have decreased or even disappeared.

Climate change is simultaneously modifying multiple abiotic and biotic factors (e.g. temperature, environmental ranges of pests and precipitation patterns) and decoupling linked factors that trigger important developmental responses (Anderson, 2016). For example, a decoupling of photoperiod and temperature may trigger phenological transitions and a mismatch between current and optimal phenotypes (Etterson and Shaw, 2001). Warm rains in early spring followed by a major frost event can kill germinating seedlings as maternal effects may have changed the timing of germination by altering the seed size and seed coat (Rodrigo, 2000; Kolesnichenko *et al.*, 2003; Lucas *et al.*, 2008). Nutrient stress and higher temperatures may result in small seed sizes that disperse far from the maternal plant and land in habitats that do not match the maternal environment (e.g. different soil type) (Ehrlen and Eriksson, 2000; Schuler and Orrock, 2012). The leaf development of most temperate tree and shrub species is highly sensitive to temperature, and leaf development generally has advanced earlier due to climate change and higher temperatures (Polgar and Primack, 2011). However, these shifts in temperature may provide unreliable cues for the initiation of leaf development. For example, temperature increases may induce leaf development before organisms can fulfil their chilling requirements (Polgar and Primack, 2011). The reliability of environmental cues on the expression of phenotypic plasticity has the potential to have a large impact on the fitness of the organism.

Phenotypic plasticity has been well documented in the shifting of phenological, photoperiod and other responses due to climate change (e.g. Bradshaw and Holzapfel, 2001; Gordo and Sanz, 2006; Byers, 2017). However, the responses of one species relative to other species in these shifts are often more ecologically important than the absolute plastic response of a single species (Westerband *et al.*, 2021). For example, large temporal and spatial plastic responses in plants and pollinators could potentially cause mismatches for successful pollinations. In Japan, flowering times tended to occur earlier over the last three decades for four *Prunus* tree species as development is strongly influenced by temperature 30–40 d before flowering. However, the appearance of the *Pieris rapae* butterfly (a proxy for potential pollinators) tended to be delayed over the past three decades as its appearance is not strongly affected by temperature and its development is most sensitive to temperature at around 15 d prior to butterfly appearance. The trends in the plant and butterfly phenologies are changing in opposite directions as they rely on different climatic cues with different temporal trends (Res *et al.*, 2008).

However, changes in environmental cues due to climate change can also be beneficial. For example, changes in spruce tree phenology and budworm phenology have increased the synchrony between their life cycles (Miller-rushing *et al.*, 2010). In addition, butterflies in the larval stage grow faster and survive better on new newly colonized host plants from climate-driven range expansions (Brachler and Hill, 2007). The degree to which plasticity benefits fitness is contingent upon how reliable environmental cues predict future environments and selection regimes. For example, a stochastic individual-based model in which phenotypes could respond to a temporally fluctuating environmental cue found that plasticity was beneficial when environmental variability was present, but environmental cues were reliable. However, when there was high environmental variation, unpredictable environmental cues reduced the population size (Reed *et al.*, 2010). The predictability and reliability of environmental cues have huge consequences for the adaptive value of plasticity.

### Environmental cues are complex

Recently, there have been calls advocating for the creation of a coherent and integrative framework for the investigation of plasticity in response to more environmental complexity, rather than single-factor experiments (Westneat *et al.*, 2019), which may influence the reliability and interpretation of environmental cues (Dore *et al.*, 2018). For example, multiple integrated cues act on biochemical pathways to influence the timing of flower opening. The production of a key molecule, COP1, involved in oscillating the circadian clock, is inhibited by light-stimulated photoreceptors, entraining flower opening via light cues to particular times of the day (Ma and Yanovsky, 2009). However, altering temperatures can also alter the timing of flowering opening by affecting a separate timing oscillator independently of the light oscillator (McClung and Davis, 2010) or by influencing temperature-sensitive photoreceptors themselves (Ma and Yanovsky, 2009). Additionally, multiple environmental cues may also be integrated over time. Environmental factors may be experienced by an organism differently in time, so exposure to one environmental factor may alter the response to a subsequent environmental factor (Snell-Rood, 2012). For example, velvetleaf seedlings exposed to low red/far-red light ratios were less responsive to a second exposure to the same environmental cue when compared to seedlings that were not earlier exposed (Weinig and Delph, 2001). New insights into plastic responses may be gained through studying the role of complex environmental cues (i.e. cues comprising multiple, distinct sensory components) (Dore *et al.*, 2018). Furthermore, when multidimensional plasticity [i.e. plastic response(s) from two or more environmental factors] is non-additive, the challenge of environmental cue reliability may be multiplied (Westneat *et al.*, 2019). The complexity of multiple, dynamic environmental cues on the expression of phenotypic plasticity and how this influences the cost–benefit paradigm of phenotypic expression is a complex but important aspect of phenotypic plasticity.

## COSTS AND LIMITS OF PLASTICITY

Costs and limits of plasticity may more broadly refer to the ‘jack of all trades is a master of none’ (Richards *et al.*, 2006). If limits and costs to plasticity did not exist, organisms should be able to produce infinite plasticity and express the superior phenotype in every environment (Grime and Mackey, 2002). Plasticity is beneficial when the organism can express superior phenotype–environment matches across more environments compared to a fixed phenotype. However, organisms may not be able to express this perfect or infinite plasticity because of the costs for the ability to be plastic or express the plastic phenotype, limits to plastic expression, or inability to reliably perceive environmental cues (Schlichting, 1986; Moran, 1992; Via *et al.*, 1995; Scheiner, 2003).

The costs of plasticity are defined as the fitness tradeoffs associated with plastic responses. In comparison, the limits of plasticity are defined when the genotypes with a plastic response cannot achieve the same phenotype as a fixed or non-plastic genotype (Dewitt *et al.*, 1998). In terms of developmental processes, the ‘developmental range’ limit is the idea that by non-plastic genotypes focusing on a specific fixed trait, they may be able to express more extreme trait values and/or traits better matched to the specific environment. It was speculated that the developmental range limit was an important limit to plasticity as the range of phenotypic expression in response to plasticity within species is often smaller when compared to trait divergence across species (Dewitt *et al.*, 1998). However, the ‘developmental range’ limit has been speculated to be the consequence of the maintenance or production costs of plasticity (Kleunen and Fischer, 2004). In addition, in plants, there is little support for the ‘developmental range’ limit. For example, in clonal herbs responding to competition, the most plastic individuals were the most successful (Kleunen *et al.*, 2000), and in plant defence studies, the most plastic individuals express the highest defence against herbivores (Morris *et al.*, 2006).

Plastic responses that have a greater cost reduce the likelihood of that response persisting within and across generations and favour the fixed average phenotype expression (Schlichting and Pigliucci, 1998). However, there is a distinction between the costs of an induced plastic response and the cost of the ability to potentially express a plastic response (Dewitt *et al.*, 1998). Expressing plastic traits themselves can be costly, for example upregulating plant chemical defences (Cipollini *et al.*, 2014). However, these trait-specific costs, or costs associated with expressing a trait, may also be experienced by non-plastic genotypes expressing the same trait. In addition, trait-specific costs are often masked by the fitness benefits of the plastic response.

However, the costs associated with the ability to be plastic (i.e. the cost of the ability to change phenotypic expression or carrying the genetic and/or sensory machinery, not the cost of the new phenotype) are often overlooked and are challenging to quantify. However, some examples are documented in the literature. In radish, the ability of a plant to mount a defence induced by predators is costly in terms of lifetime fruit mass production (Agrawal *et al.*, 2002). In addition, in *Trifolium repens*, no costs to plasticity in terms of petiole length and leaf area were detected when plants were exposed to homogeneous shade. However, genotypes grown under high light conditions experienced significant costs for the ability to express plasticity in petiole length and leaf area when exposed to shade conditions (Weijschede *et al.*, 2006). A meta-analysis of plastic responses in plants and animals concluded that the costs for the ability to be plastic are small and infrequent but tend to be greater in stressful conditions, indicating that the costs increase in the context of competition, herbivory or resource limitation (Van Buskirk and Steiner, 2009). For example, the cost to mustard plants of adjusting leaf area is typically only present in low light (Steinger *et al.*, 2003). However, a simple model of intrinsically costly plasticity does not fit well with the huge complexity in which plants adjust in response to abiotic and biotic stresses. Therefore, simply identifying negative correlations between plasticity and realized fitness may not be sufficient to test the existence of costs to plasticity. The analysis must occur at many levels and examine the underlying metabolic mechanisms and their consequences for whole plant performance and adaptation in various environments (Murren *et al.*, 2015).

The ordinary least squares estimator has been a commonly used model to estimate the costs of plasticity, but can provide poor estimates in the presence of multicollinearity and outliers in the data. Recently, a robust ridge estimator model, less affected by multicollinearity and outliers compared to the ordinary least squares model, has been proposed to estimate the costs of plasticity. The robust ridge estimator detected costs of plasticity that were severely and incorrectly underestimated by the ordinary least squares estimator (Michimae *et al.*, 2018). Evaluating the costs of plastic responses can provide important insights into the fitness landscape of a plastic response.

The costs for the ability to be plastic can be incurred via the expenditure of energy to gain reliable information about the environment, through assessing multiple environmental cues, or investing resources to improve the assessment of environmental cues. This may include when a plastic organism requires sensory and regulatory machinery that an organism expressing a fixed phenotype does not. For example, there is a cost associated with the sensory machinery that regulates the ethylene growth response, the ethylene receptor proteins on the cell membrane (Jones, 1994). The cost associated with producing the receptor proteins is a cost of plasticity that a fixed phenotype insensitive to ethylene may not have to produce. One way of assessing the cost of carrying additional genetic machinery is to evaluate the genome size. For example, the repeated evolution of aneuploidization and variation in transposable element load suggests fitness benefits from removing specific non-coding genetic sequences (Hu *et al.*, 2011). However, in most cases, the cost of carrying unexpressed genes, regulatory elements or sensory machinery may be negligible. For example, in *Daphnia* genotypes that are considered to be specialist (narrow tolerance) or plastic (broad tolerance) to salinity tolerance, the analysis of the plastic expression of over 900 genes revealed no differences in the amount of transcription, protein length or ATP production (Latta *et al.*, 2012). The costs of the ability of an organism to be plastic in many cases are considered negligible in plants (but may be considerable for organisms with brains or sophisticated immune responses) (Murren *et al.*, 2015). Our understanding of the compounds, signalling cascades and metabolic pathways involved in stress response has grown exponentially in recent years. In addition, technological advancements in molecular methods enable individual traits to be isolated, and their fitness determined. Disentangling the costs of the ability to be plastic from the costs of the actual expression of the phenotype by evaluating molecular machinery and regulation of phenotypic plasticity is an important but often overlooked aspect of plasticity research.

Additional genetic costs to plastic responses are also important to consider. Phenotypic plasticity may be expressed because structural genes or gene products are directly affected by environmental cues (i.e. allelic sensitivity), or regulatory genes are affected by the environment and influence the expression of structural genes (Via *et al.*, 1995). However, genes associated with plastic responses may be linked to genes associated with reduced fitness; for example, genes associated with the plastic response may have negative pleiotropic effects on other traits, or epistasis could cause regulatory loci associated with the plastic response to modify other genes (Dewitt *et al.*, 1998; Schneider and Lynch, 2020). Pleiotropy, epistasis, linkage and other genetic factors in plasticity expression are rarely studied, but may often be confounding factors when analysing the influence of plastic responses on fitness.

The costs of plasticity are not mutually exclusive, and they contribute in varying degrees depending on the specific environment. The costs of plasticity are often difficult to detect and measure (see meta-analyses by Kleunen and Fischer, 2004; Van Buskirk and Steiner, 2009). Past selection patterns may impede our ability to detect the costs of plasticity. Theoretically, there should be a strong selection against the costs of plasticity, and gradually responses and mechanisms should evolve to reduce the costs of plastic responses or evolve to reduce the expression of costly plastic responses (Murren *et al.*, 2014).

Furthermore, the cost of canalization may reflect plasticity costs, but for a trait not measured or considered (Van Tienderen, 1991). For example, individual traits, including specific leaf area, root length and leaf nitrogen content, may all express plasticity to maintain phenology or a stable yield. In this case, if yield or phenology is measured, a cost of canalization rather than plasticity may be detected. A meta-analysis revealed that in 71 % of cases testing costs of plasticity, costs of plasticity and canalization were equally frequent and relatively mild (Van Buskirk and Steiner, 2009), which suggests that in many cases we are not measuring the appropriate traits as proxies for plastic responses. Many speculate that measuring biological complexity more thoroughly, including more complete evaluations of fitness and characterizing diverse cues that induce plasticity, may enable us to better understand plasticity (Westneat *et al.*, 2019). It is important to consider which traits are expected to vary in expression to maintain constant and stable fitness-related traits across fluctuating environments.

## COST–BENEFIT RATIO

A critical challenge organisms face in heterogeneous environments is assessing the cost–benefit ratio, or whether or not the ‘cost’ of expressing a plastic response outweighs its potential benefits. Generally, natural selection should favour organisms that can express phenotypes with a low cost–benefit ratio (West-Eberhard, 2003; Sultan, 2015). For example, the cost–benefit ratio of how a plant responds to a heterogeneous distribution of phosphorus in the soil depends on the size of the nutrient patch or how limiting phosphorus is for growth. If the size of the nutrient patch is too small, the investment of carbon and nutrient resources in tissue construction and maintenance of lateral root proliferation may not pay off for the uptake of relatively little phosphorus. However, a large nutrient patch may be an environmental signal to induce a plastic response that enhances phosphorus capture and, subsequently, plant fitness. In addition, the lifetime seed projection of *Nicotiana attenuata* plants treated with jasmonic acid was reduced compared to untreated plants when herbivores were absent. However, when treated plants were exposed to moderate or high levels of herbivory, the fitness benefits exceeded the cost of jasmonic acid elicitation (Baldwin, 1998). The cost–benefit ratio of jasmonic acid elicitation in *N. attenuata* plants depends on the magnitude and severity of the risk of herbivory.

In natural and agro-ecosystems, plants may be exposed to multiple, simultaneous or successive stresses. For example, in growth environments with terminal drought, seeds are planted in the moist topsoil, but drainage, evaporation and plant water uptake results in the topsoil progressively drying and therefore becoming harder while resulting in greater water availability in deep soil strata (Lynch, 2013; Lynch *et al.*, 2014). Tissue construction and maintenance demand significant resources (Nielsen *et al.*, 2001). However, the investment of carbon and nutrient resources in the construction and maintenance of tissues early in plant growth may limit the opportunity to construct other tissues. For example, in roots, if carbon and nutrient resources are invested early in the growth season for root proliferation in the moist topsoil, this limits the opportunity for the construction of roots later in the growth season in deeper soil strata where softer soils and deep water are likely to be located (Schneider and Lynch, 2020). Numerous cost–benefit ratios in different environmental scenarios have been studied in the literature, ranging from mycorrhizal associations, root depth, the development of root hairs to leaf shape and size (Koide *et al.*, 1988; Madsen, 1991; Guswa, 2008; Zhu *et al.*, 2010).

## APPROACHES, CONSIDERATIONS AND PROMISING RESEARCH DIRECTIONS IN STUDYING PLASTICITY

Typically, plastic responses are quantified experimentally by growing a single genotype in two or more different environments with a contrasting environmental factor (e.g. temperature, pH, nutrient level, competitor, pests). This experimental approach goes back over 75 years (see references in Sarkar, 2004). Various approaches have been used to study plastic responses of plants, animals and their interactions. Empirical and modelling approaches are most common. However, historical records, including herbarium specimens, and long-term field observations have also been used (Byers, 2017). Considerations in experimental, modelling and statistical approaches are needed to test proposed individual- and population-level consequences of plasticity (Wennersten and Forsman, 2012) and the influences of plasticity on fitness, its costs and genetic mechanisms (Forsman, 2015). However, the quantification and interpretation of phenotypic responses and their evolution are often misguided due to limitations and pitfalls of experimental designs, statistical tools and analytical approaches. Several approaches and considerations are highlighted below for studying phenotypic plasticity.

### Selection of traits and environments to study

Studies of plasticity are typically focused on specific traits with established functional significance. However, this poses a risk with a reductionist approach that this biased selection may overestimate the importance and adaptive value of plasticity. To get a balanced view of the function and value of plasticity and its evolutionary dynamics, it is important to identify environmental factors that do not elicit plastic responses and environmental conditions in which plasticity may be maladaptive or less beneficial (Forsman, 2015; Westerband *et al.*, 2021). In addition, considering multiple interacting plastic traits (i.e. multivariate plasticity) should also be an essential component of plasticity studies. The cause and consequences of these plastic interactions between different traits have received relatively little attention; however, recently, a new conceptual framework has been proposed to understand why organisms should have multiple responses and how these responses can influence each other (Nielsen, 2022). Plasticity in one trait may alter the phenotype of another trait by changing the cue received by the organism or the response to that cue and may result in synergistic, antagonistic, complementary or other effects (Nielsen, 2022).

Designing treatments requires consideration of temporal and spatial distribution of variation in nature. For example, instead of varying the factor mean in alternative fixed treatments, it may be more ecologically meaningful to vary the timing, duration, range or periodicity of environmental conditions (Miner and Vonesh, 2004). The environmental treatments need to elicit plastic responses while simultaneously being controlled with enough precision to interpret responses to specific environmental cues (Miner *et al.*, 2005). The environmental cue that induces a plastic response may appear relatively straightforward. For example, the amount of photosynthetically active radiation has been demonstrated to induce plastic responses (Klem *et al.*, 2015). However, plastic responses due to the amount of available photosynthetically active radiation have many nuanced aspects, including the distribution of available light, which may also influence plasticity (Franklin, 2008; Ballaré, 2009). Canopy structure influences the amount and diurnal distribution of light in a forest, and diurnal responses to light are often overlooked in favour of the total amount. However, Wayne and Bazzaz (1993) demonstrated that birch seedlings provided with the same total, reduced daily photosynthetically active sunlight had different plastic responses depending on whether the light was provided at a consistent level throughout the day or very low light with brief intervals of full intensity (Wayne and Bazzaz, 1993). Temporal treatments can also reveal how rapidly an organism can adjust to relevant functional traits, revealing information about timing differences and duration of responses between genotypes.

**Fig. 2.**
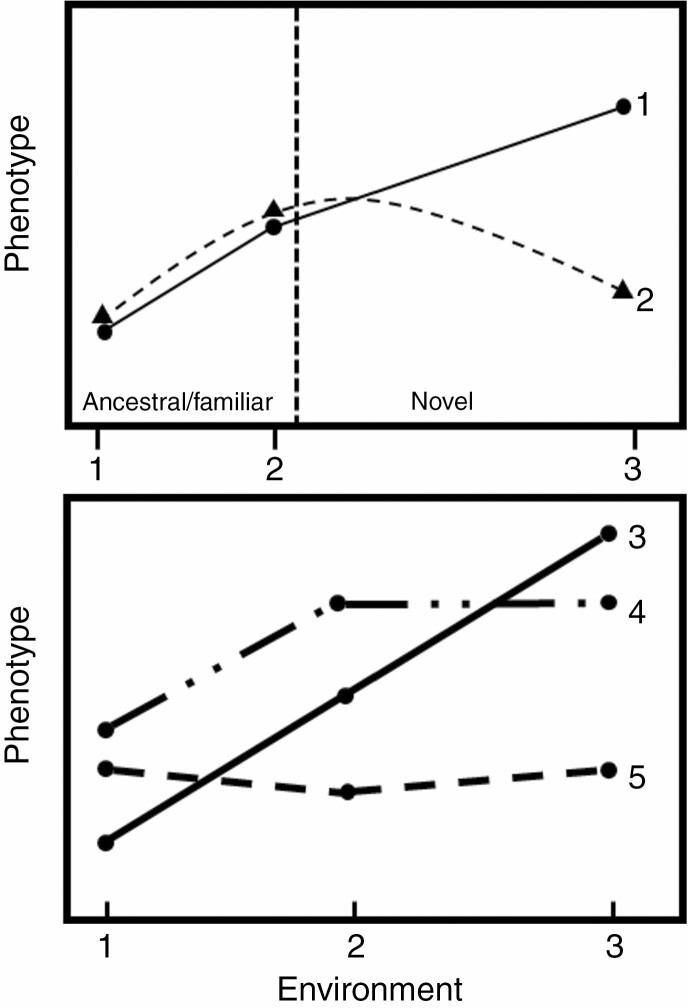
(A) Cryptic genetic variation. In ancestral and familiar environments, genotypes 1 and 2 do not display genotype by environment interactions as their reaction norms are similar and parallel. However, genotypes 1 and 2 diverge in evolutionarily novel environments, demonstrating hidden reaction norms and cryptic genetic variation. (B) The norms of reaction for three genotypes in three environments. For a specific trait, each genotype is phenotyped in each environment and these points are joined to form a reaction norm. (3) a genotype varies continuously and linearly, (4) a genotype response with a few discrete alternatives (i.e. polyphenism), (5) a genotype is fixed or relatively canalized across environments. Genotypes 3, 4 and 5 display genotype by environment interactions as their reaction norms are not parallel. Modified from Sultan (2015).

The careful selection of the environment may also reveal ‘hidden’ plasticity. Plasticity can be expressed in hidden reaction norms or ‘cryptic plasticity’, which occurs when plastic responses are not expressed in the ancestral environment, but an environmental shift reveals potential for a plastic response (Table 1). In scenarios revealing hidden reaction norms, individuals must be subjected to novel environments typically beyond the environmental limits to which it is adapted (Fig. 2A). New approaches, like *np*^*2*^QTL, not only enable the mapping of genetic loci associated with plasticity but also enable the estimation and testing of the slope and curvature of reaction norms to uncover cryptic genetic variation of plastic responses (Ye *et al.*, 2019). The selection of the range and severity of environmental factors is an important consideration to understand plastic responses.

When an organism integrates multiple environmental factors into expressing phenotypic plasticity (i.e. multidimensional plasticity), the molecular mechanisms, adaptive value for fitness and its implications for evolution may vary compared to plasticity in response to a single environmental factor. For example, in Mediterranean oak, precipitation and temperature regimes play an important role in the evolution and expression of coordinated multivariate phenotypic plasticity in several morphological traits (Sole-Medina *et al.*, 2022). Multidimensional plasticity cannot be interpreted by simply scaling up univariate plasticity to more environments as these interactions often result in unexpected expression of plasticity. A more comprehensive characterization of the complexity of environments that induce plasticity may reveal insights into the interaction between genes and the environment. Multidimensional plasticity has been rarely considered in plasticity models and its influence on evolution and speciation, yet it may greatly influence the nature of speciation and adaptation to environmental change (Westneat *et al.*, 2019; Morel-Journel *et al.*, 2020).

### Interpretation of plastic responses

The interpretation of plastic responses is important. Responses positively associated with fitness are typically functionally adaptive; however, because environmental factors influence both traits and fitness, these analyses and associations can be biased (Stinchcombe *et al.*, 2002; Winn, 2004). In scenarios where plastic responses are irreversible or can be experimentally augmented, the utility for plant fitness can be tested by directly comparing plastic and non-plastic responses within environmental treatments (Schmitt *et al.*, 2003). However, often plastic responses are interpreted as adaptive based only on ecological or functional factors (Bonser, 2021): for example, herbivore-induced chemical and structural defences or partitioning or structural changes to tissues that help optimize a limiting resource (references in Sultan, 2015). We need to better understand the fitness landscape of plastic responses (i.e. how traits influence performance in several different environments and combination with several different plant traits). We must also understand the conditions that maintain or promote plastic responses and distinguish between adaptive, neutral and maladaptive responses in regard to fitness.

### Selection and evolution of plasticity

The selection on and evolution of phenotypic plasticity has been widely studied (see references in Van Tienderen, 1991; Arnold *et al.*, 2019*b*; Sommer, 2020; Pfennig, 2021)). The degree of environmental variation has been shown to influence the magnitude and frequency of plastic responses. For example, species with high dispersal rates, and therefore greater spatial variation, tend to display greater developmental plasticity (Hollander, 2008). However, in many cases, environmental variation may explain little to no variation in plasticity and only account for or contribute a portion of the evolution of plastic responses (Karban and Nagasaka, 2004). A high degree of plasticity is expected to evolve in predictable environments, and reduced plasticity is expected to be expressed in environments that fluctuate less predictably as these plastic responses may not match future selective pressures (Gavrilets and Scheiner, 1993; Botero *et al.*, 2015; Tufto, 2015). However, a recent meta-analysis highlighted the need to more precisely characterize plasticity, as these predicted trends were only true for allocation traits (which may be a form of passive plasticity) and non-climatic factors were more strongly associated with plasticity than climatic factors (Stotz *et al.*, 2021).

Recently the importance of considering adaptive plasticity in breeding programmes has been highlighted to address crop responses to increasing environmental variability (Larson *et al.*, 2014; Sadras and Richards, 2014; Sadras and Denison, 2016; Lobet *et al.*, 2019; Monforte 2020; Schneider and Lynch, 2020). Understanding how plasticity evolved and was selected during domestication may provide important insights in this context (Larson *et al.*, 2014). The onset of domestication was accompanied by strong genetic bottlenecks which may have influenced the persistence of plasticity (see references in Matesanz and Milla, 2018). Several recent studies provide mixed evidence and no robust pattern for the effects of domestication on phenotypic plasticity, which is difficult to interpret as it depends on the adaptive value of the individual trait combinations and their interaction with the environment. For example, during the selection of modern temperate maize, genomic regions associated with genotype-by-environment interactions and plasticity were neither directly nor indirectly selected to increase plant performance and yield stability (Gage *et al.*, 2017). However, several genomic loci have been identified that are associated with plasticity in yield components in maize hybrids, and evidence suggests that they may have been selected for during the tropical–temperate adaptation process (Liu *et al.*, 2021). Annual phlox and several rice genotypes showed no difference in plasticity in root and shoot morphological traits between wild and domesticated cultivars (Schlichting and Levin, 1988; Shimizu *et al.*, 2010). Wild barley germplasm exhibited greater within-plant root plasticity to soil nutrient concentration and distribution when compared to domesticated cultivars (Grossman and Rice, 2012). In barley, selection against thermal clock plasticity occurred during domestication (Prusty *et al.*, 2021). In a large study involving domesticated and wild chard, cabbage, sunflower, tomato, durum wheat, maize and pea, domesticated genotypes outperformed wild genotypes in favourable conditions but suffered greater reductions in performance in stressful conditions. However, plasticity was expressed in wild and domesticated genotypes to similar degrees and varied by trait (Matesanz and Milla, 2018). This evidence highlights the complex nature of plastic responses and their interaction with the environment to produce adaptive responses and the potentially high costs of the expression of plasticity.

Examining plastic responses of wild crop ancestors and landraces to stresses they may encounter in their native environments may provide insight into potentially adaptive plastic responses for fitness. For example, several wheat landraces may be adapted to dry and hot conditions (Morgounov *et al.*, 2021). Therefore, plastic responses of these wheat landraces to drought and heat may provide insight into potentially adaptive plasticity that may not be present in modern cultivars. Modern cultivars may not have been subjected to these constraints in evaluation and selection in breeding programmes in modern agroecosystems. Landraces and wild crop ancestors may be an important resource in characterizing plasticity and interpreting its adaptive value. In addition, comparing genotypes or populations adapted to different environments may help identify factors driving the evolution of plastic responses and provide insight into how plasticity can contribute to evolutionary differentiation within species (e.g. Nunes *et al.*, 2014).

### Integrated approaches are needed to study plasticity

In studies focused on plasticity, typically, environmental cues are manipulated, and phenotypic responses (morphology, metabolic rates, epigenomic modifications, etc.) are measured. Interdisciplinary approaches involving molecular genetics, physiology and soil science that integrate these layers of plastic responses may provide insights into cue perception, transduction and phenotypic response (Morel-Journel *et al.*, 2020). For example, in plant responses to insect herbivores, layered responses occur at the levels of phytohormone signalling, metabolism, growth rate, epigenetic signatures, metabolites and phenology (Schuman and Baldwin, 2016). The study of phenotypic plasticity should include research on these different scales to understand plant responses to critical environmental cues.

Many aspects of plasticity may be useful to examine *in silico*. Several models have been developed to test the adaptive value of plasticity, the importance of reliable environmental cues, population persistence, cost–benefit ratios of trait expression, interpretation of different types of phenotypic variation and selection on plasticity (e.g. de Jong, 1995; Hollander, 2008; Reed *et al.*, 2010; Wang *et al.*, 2013; Xue and Leibler, 2018; Filipe and Kyriazakis, 2019; Klingenberg, 2019; Kellermann *et al.*, 2020; Scheiner *et al.*, 2020). In addition, an emerging modelling approach, ΔTraitSDM (i.e. a species distribution model examining phenotypic trait variation), includes phenotypic plasticity and local adaptation of fitness-related traits measured across geographical scales to enable novel insights into population sensitivity to climate change (Benito and Robson, 2019). These models will be useful in understanding plasticity across biological scales, its molecular mechanisms and adaptive value because the complex nature of plasticity across traits and environmental ranges make many combinations empirically impossible to test.

### Genetic and epigenetic approaches and considerations

Since organisms live and have evolved in dynamic environments, a wide range of molecular mechanisms have evolved to help them buffer against environmental perturbations (Frankel *et al.*, 2010; Levy and Siegal, 2012; Klupczyńska and Pawłowski, 2021). Several studies have characterized molecular responses that mediate plasticity (as reviewed in Nicotra *et al.*, 2010; Lachowiec *et al.*, 2016). However, many of these mechanisms are still unknown or poorly understood due to the wide range of environmental signals and phenotypic responses. Recently, a framework has been proposed to study the genetic basis of phenotypic plasticity and highlights quantitative genetics and gene hub approaches (Laitinen and Nikoloski, 2019). Furthermore, approaches have been developed that integrate genetic mapping, evolutionary game theory and predator–prey theory (Yang *et al.*, 2021).

Plasticity in gene expression can be examined on an individual gene by gene basis, collapsing genes into co-regulated networks (e.g. weighted correlation network analysis), or by examining expression pattern shifts across the transcriptome such as gene regulatory networks (e.g. Kenkel and Matz, 2016). Recently, a reaction norm framework has been proposed to aid in the interpretation of gene expression as a plastic trait (Rivera *et al.*, 2021). However, it is important to consider many nuances in linking gene expression to phenotypes. Transcriptomic studies can provide real-time insights into plastic responses. However, alternative mechanisms such as the onset of stress response, life history, environmental history and microbial influences could also influence the gene expression patterns before, during and beyond the scope of the experiment. Gene expression is inherently dynamic, and sampling should aim to span the time frame of the plastic response to the specific environmental condition to more carefully capture changes in gene expression (Rivera *et al.*, 2021). Furthermore, careful consideration in which tissues are studied is important as environmental cues are often perceived in a particular organ different from (or in addition to) where the responses are expressed.

Several other genetic approaches have been successful in unravelling the complex genetic architecture of plasticity. For example, an integrated analysis of genomic responses of flowering time to the environment was used to identify hidden patterns and factors underlying plasticity in plants from the field. A systematic genome-wide performance prediction framework was established through genotype-specific reaction norm parameters or genome-wide marker-effect continua that exploited genomics, environmental profiling and performance information. Phenotypic plasticity was attributed to specific genes and influenced by photothermal time and gene–gene interactions (Li *et al.*, 2018). Systems evolutionary game networks have also been used to interpret gene interdependence, predict how various environmental factors influence transcriptional co-regulation, and provide insight into the mass, energetic or signal basis that drives different gene interactions. The systems evolutionary game network is a computational model that reconstructs temporal variation in gene networks and track real-time changes in network architecture underlying plastic responses. This approach was used to identify and characterize gene co-regulation that modulates the time trajectories of poplar tree responses to salt stress (Jiang *et al.*, 2020). The molecular mechanisms underlying plastic responses are generally not well understood, but new frameworks and technological advancements will enable new insights into this area.

To understand how phenotypic plasticity works mechanistically, epigenetic mechanisms, including DNA methylation, may be a key factor in linking environmental cues and gene expression. Phenotyping large association panels and using genome-wide association mapping, common garden or reciprocal transplant studies may be useful to assess phenotypic variation and partitioning variation into genetic and environmental components. However, these approaches often fail to identify genetic markers associated with plastic responses, and epigenetic mechanisms and variation could account for some of this ‘missing’ variation. Recent technological breakthroughs in the field of epigenetics (e.g. Perrone and Martinelli, 2020; Lloyd and Lister, 2022) and the development of statistical methods, including environmental association analysis and epigenome-wide association studies, have enabled the study of the relationship between epigenetic variation and plastic responses (Moler *et al.*, 2018). A better understanding of how plants store information about life history and the environment will enable us to better manipulate plasticity for further studies or breeding efforts to introduce novel stable or plastic phenotypes into crops.

### Quantifying plastic responses

**Fig. 3.**
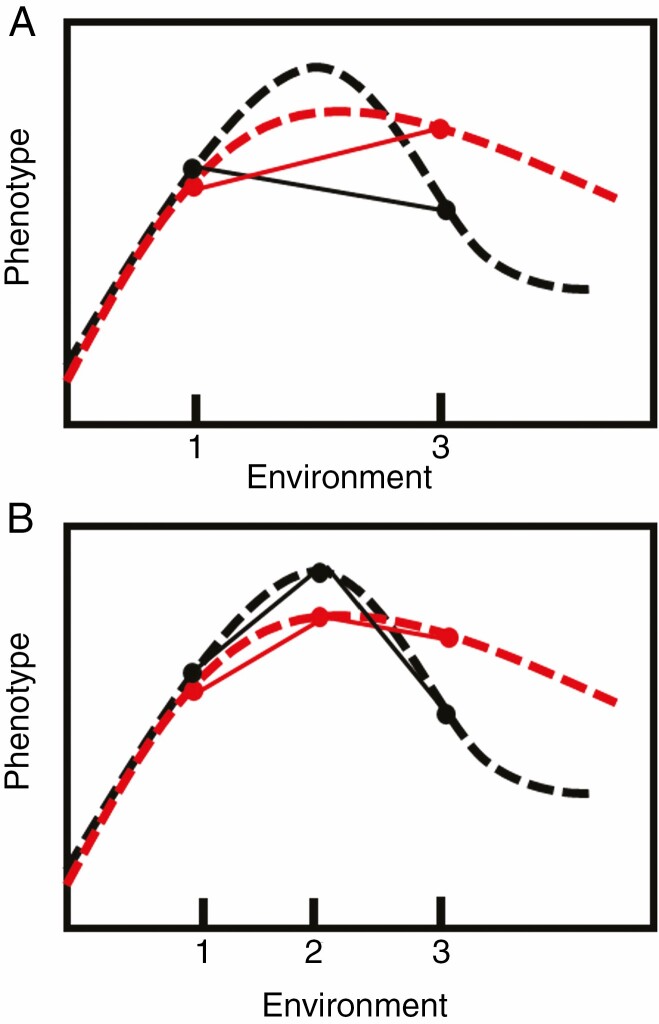
Multiple environments are needed to accurately characterize reaction norms. (A) Description of reaction norms (dashed lines) of two genotypes using only two environmental points (solid lines and points); (B) description of reaction norms using three environments captures much more of the underlying reaction norm shape. Fitting a linear line to two environments may severely over- or under-estimate the plastic response. Modified from Arnold *et al.* (2019*a*, *b*).

Many approaches have been used to quantify phenotypic plasticity, such as the formulation of plasticity indices (Valladares *et al.*, 2006; Sadras *et al.*, 2009; Murren *et al.*, 2014) and characterization of reaction norm shape (Izem and Kingsolver, 2005). However, many of these approaches assess the response and variation of plasticity in multiple steps, for example by first analysing variation at the genotype level by extracting indices and then analysing the indices. However, this ‘statistics-on-statistics’ approach makes biological interpretation challenging (Morrissey and Liefting, 2016), particularly when traits are expressed as a proportion or a ratio to minimize allometric effects. In addition, the over-simplification of reaction norms across just two environments may obscure key elements of phenotypic plasticity to changing environments and may ultimately impede a comprehensive biological understanding of plastic responses (Chevin and Lande, 2011; Arnold *et al.*, 2019*a*) (Figs 2B and 3). If reaction norms are non-linear, choosing two arbitrary points on an environmental range may not accurately represent the plastic response and may severely under- or over-estimate the phenotypic response. Furthermore, some reaction norms may have different shapes for different traits of the same species across the same environmental range (e.g. Vitasse *et al.*, 2010; Arnold *et al.*, 2019*a*) and among species in the same trait and range of environments (e.g. Schou *et al.*, 2017). Many commonly used metrics to quantify phenotypic plasticity present several limitations for application and interpretation, including comparing multiple environments, constraints of non-normal data and comparisons between species (Valladares *et al.*, 2006). In a recent meta-analysis, some of the most commonly used plasticity estimators (i.e. plasticity index, slope of reaction norm and relative distances plasticity index) were demonstrated to generate different plasticity rankings between genotypes, populations and species in a considerable proportion of studies (16 %) (Wang *et al.*, 2022). The most appropriate method to quantify plastic responses depends on the trait, genotype and/or species, as well as the number of environments and particular research questions. Several methods for quantifying plasticity and selection on plasticity have been reviewed (Valladares *et al.*, 2006; Arnold *et al.*, 2019*a*, *b*) and their drawbacks have been highlighted (Valladares *et al.*, 2006; Pélabon *et al.*, 2020). Below, several analytical tools and approaches for quantifying phenotypic plasticity are highlighted focusing on the potential to analyse multiple traits and environments.

If plasticity is considered a trait, then the variance of the genotype-by-environment interaction is a measure of the variance in the plastic response over the measured genotypes (Marais *et al.*, 2013), but not for individual genotypes. The coefficient of the plastic response of the trait can be derived as the dimensionless slope of the linear regression between the trait of a single genotype in a specific environment and the mean of all genotypes in that environment, also known as a Finlay–Wilkinson regression (Finlay and Wilkinson, 1963). Therefore, a slope >1 indicates above-average plasticity, and a slope <1 indicates below-average plasticity (Sadras *et al.*, 2009). Finlay–Wilkinson regression has been used to dissect the genetic architecture of phenotypic plasticity in plants. For example, the slopes and mean residual deviation of Finlay–Wilkinson regression lines were used as responses modelled by genome-wide association analysis to detect loci associated with genotype-by-environment interactions in maize yield (Gage *et al.*, 2017). In addition, estimates of plasticity from the Bayesian formulation from the Finlay–Wilkinson regression have also been used in genome-wide association analysis in maize to identify loci associated with plasticity for yield components and shoot architecture (Kusmec *et al.*, 2017).

The AMMI (additive main-effects and multiplicative interaction) model combines ANOVA (analysis of variance) for the main environment and genotype effects with a principal component analysis of genotype-by-environment interactions. AMMI incorporates both additive and multiplicative components into an integrated least-squares analysis. Subsequently, the genotype and environment metrics obtained from AMMI can be represented into a biplot, which allows for the interpretation of differences in phenotypic plasticity and adaptation patterns. Similarly, the factor analytic (FA) model assumes that genotype-by-environment may be explained by latent variations and examine patterns of genotype-by-environment interactions and stability across environments (Elias *et al.*, 2016; Xie *et al.*, 2021). Both AMMI and FA were used to quantify plasticity in root and yield traits in rice in response to drought (Xie *et al.*, 2021). However, the FA model allows for both fixed and random factors and assumes genotypes are random effects, while the AMMI model assumes experimental error is homogeneous across environments and genotypes are fixed effects.

Instead of quantifying plasticity at the level of the whole organism or single traits, several phenotypic dimensions can be simultaneously modelled and compared using multivariate statistical analysis or composite measures of plastic responses based on trait averages or dimension-reducing techniques [e.g. principal component analysis (Carroll and Corneli, 1991; Nussey *et al.*, 2007; Forsman, 2015)]. The multivariate plasticity index (i.e. the Euclidian distance between scores of a canonical variate analysis) enables multiple traits to be condensed into a single parameter, allowing for the comparisons of species or genotypes in a systematic way (Pennacchi *et al.*, 2021). Random regression mixed models have been proposed as a method to describe phenotypic plasticity over multiple environments at both a population- and genotype-level response (Arnold *et al.*, 2019*a*). Random regression mixed models also have the potential to consider multivariate responses, multiple environmental variables and quantitative genetic analysis. For example, additional experimental covariates can be incorporated into the model as fixed (e.g. number of leaves) or random (e.g. replications) effects. The covariance between genotype-specific intercepts and slopes may be estimated and indicates whether genotypes with greater trait values have more or less phenotypic plasticity (Nussey *et al.*, 2007; Arnold *et al.*, 2019*a*). Random regression mixed model analysis fits individual reaction norms and has the flexibility to model a variety of shapes of linear and non-linear responses to different environments. A single mixed model analysis is flexible enough to accommodate unbalanced designs and different reaction norm functions and avoids the drawbacks of multistep approaches (Arnold *et al.*, 2019*a*). Multivariate random regression models have been used to analyse plastic responses of multiple traits and the selection on plasticity in a variety of species and environments (Arnold *et al.*, 2019*b*).

The numerous indices and metrics used to calculate and quantify plasticity generate various outputs, which complicate the analysis and interpretation of plasticity, particularly in comparative studies and multiple environments. Particular attention should be paid to quantifying phenotypic plasticity with non-normal or non-linear data and the ability to interpret plastic responses between genotypes and populations across multiple traits and environments. The most appropriate method may also depend on the specific characteristics of plasticity (e.g. continuous/discrete, short/long duration) (Table 1) expressed in the focal trait(s).

## CONCLUSIONS

The ubiquitous nature of plasticity infers that it offers an evolutionary or fitness advantage (Nijhout, 2003; Palacio-López *et al.*, 2015). Phenotypic plasticity allows organisms to modify their phenotype to match their current environment, including in fluctuating or novel environments, which may be an evolutionary advantage. Phenotypic plasticity can be passive, instantaneous, delayed, continuous, discrete, permanent, reversible, adaptive, maladaptive and transgenerational. Virtually any abiotic or biotic factor can induce plasticity and may result in adaptive phenotypes to harmful susceptibilities. To reliably classify traits, genotypes or populations as non-plastic is challenging because it is empirically impossible to confirm that phenotypic variation is not influenced by any environmental cues because the focal trait, genotype or population may show a plastic response to environmental cues or ranges that have not been investigated (West-Eberhard, 2003; Forsman, 2015)

**Fig. 4.**
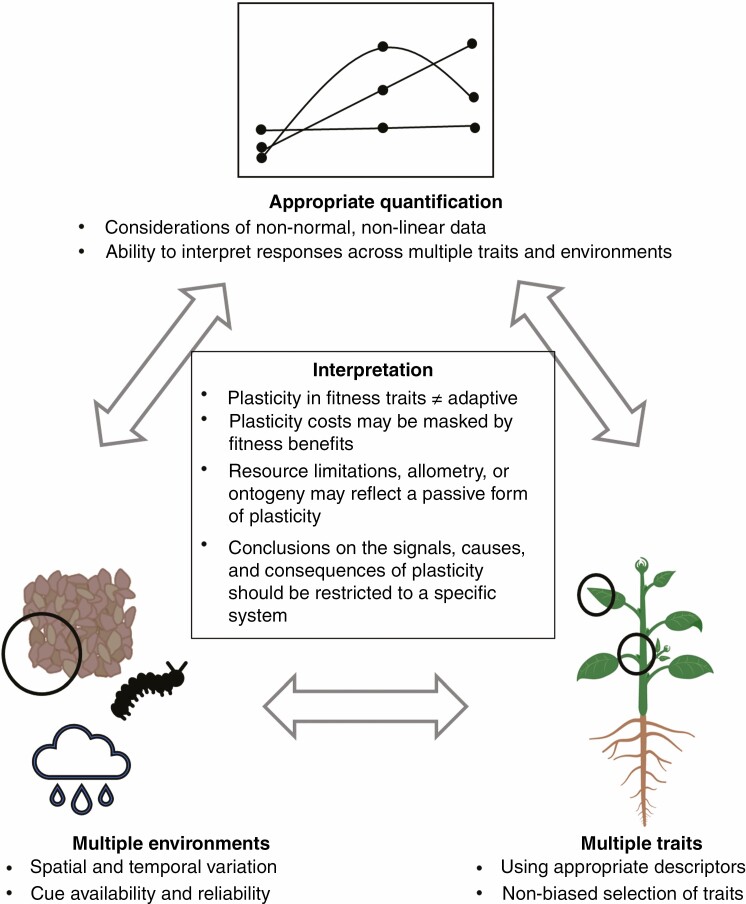
Key considerations for the study of phenotypic plasticity. Multiple environmental factors and factor levels are needed to examine plastic responses in multiple traits. The quantification method of plastic responses must be carefully considered as many methods generate different outputs, which complicate the analysis and interpretation of plasticity, particularly in comparative studies and multiple environments.

For individual traits in individual genotypes, the ideal description of phenotypic plasticity would be a predictive map outlining functional relationships of the individual’s genes and growth environment to their phenotypic expression (Matsui and Ehrenreich, 2016). However, a wide range of traits are influenced by a wide range of environmental cues that are impacted by plant development, duration and intensity of the cue, the spatial location of the cue, and its interaction with other traits. Accessing and understanding the systems biology of plasticity will require combining genetic mapping, metabolomic, transcriptomic and proteomic approaches from the same genotypes exposed to multiple environments. These complex interactions are challenging to study and understand (Fig. 4).

Our understanding of phenotypic plasticity is hampered by imprecise and ambiguous terminology as terminology is often used differently depending on the observer’s perspective and biological context. However, this highlights the value of better defining the concept of plasticity and using more precise terminology when describing plasticity. Key characteristics to define and specify types of plasticity are outlined in Table 1 and will be helpful in describing and characterizing these phenotypic changes. In addition, we should take care and consideration into how we interpret plasticity. Conclusions regarding the signals, causes and consequences of plastic responses must be restricted to the particular system studied, including the specific phenotypic dimensions, aspects of plasticity and environmental cues, and not extended into general statements regarding plasticity (Forsman, 2015).

Due to both selective histories and constraints that may have evolved, genotypes will vary with respect to the environmental cues that induce plastic responses. Environmental cues induce plastic responses across biological scales, and the adaptive value of plasticity often partly depends on the reliability of environmental cues to predict future environments. In addition, the expression of plasticity often incurs a cost, which can be attributed to the cost of the phenotype or the ability to be plastic. The costs associated with plasticity are challenging to study as fitness benefits often mask them, but they are an important aspect to consider in interpreting the adaptive value of plasticity.

Studying phenotypic plasticity in plants involves many disciplines, including genetics, entomology, soil science, physiology and evolution. The field of plasticity is a rich field for collaboration, linking traditionally distinct approaches to molecular mechanisms, evolutionary consequences and physiology. A better understanding of phenotypic plasticity will impact all fields of biology. It will require researchers to confront the fact that most phenotypes result from interactions between genes and the environment and that plastic responses are ubiquitous rather than the exception. Many aspects of plasticity are still unexplored, and future research will provide important insights into how organisms develop, function, interact and evolve.
